# Value of the Systemic Immune-Inflammatory Index (SII) in Predicting the Prognosis of Patients With Peripartum Cardiomyopathy

**DOI:** 10.3389/fcvm.2022.811079

**Published:** 2022-02-17

**Authors:** Yuan Zhang, Wenzhao Liu, Huaitao Yu, Zhen Chen, Chunmei Zhang, Yun Ti, Peili Bu

**Affiliations:** The Key Laboratory of Cardiovascular Remodeling and Function Research, Chinese Ministry of Education, Chinese National Health Commission and Chinese Academy of Medical Sciences, State and Shandong Province Joint Key Laboratory of Translational Cardiovascular Medicine, Department of Cardiology, Qilu Hospital, Cheeloo College of Medicine, Shandong University, Jinan, China

**Keywords:** peripartum, cardiomyopathy, heart failure, prognosis, systemic immune inflammatory index

## Abstract

**Background:**

Peripartum cardiomyopathy (PPCM) is a potentially life-threatening complication of pregnancy. The identification of early prognostic markers in patients diagnosed with PPCM is very important. The systemic immune-inflammation index (SII) is a new inflammatory biomarker, and the aim of this study was to evaluate the prognostic value of SII in patients with PPCM.

**Methods:**

A total of 61 patients with PPCM who were admitted in our hospital from 2015 to 2020 were retrospectively analyzed in this study. The follow-up period of all patients was at least 6 months after diagnosis. Recovery of left ventricular (LV) systolic function was defined as the presence of left ventricular ejection fraction > 45%. The second endpoint was defined as composite adverse cardiac events, including cardiac death or hospitalization due to worsening heart failure. Univariate and multivariate logistic regression analysis were used to determine the independent predictors of non-recovery of LV systolic function. The receiver operating characteristic (ROC) curve analysis was used to establish a cut-off level of SII value to predict persistent LV systolic dysfunction.

**Results:**

The follow-up duration was 40.5 ± 16.3 months. Among the 61 patients, 43 patients showed left ventricular recovery and 18 patients did not at the last follow-up visit. The baseline SII levels were significantly higher in the non-recovery group (*P* < 0.05). Multivariate logistic regression showed that the SII and left ventricular end-diastolic dimension (LVEDD) were independent predictors of persistent LV systolic dysfunction (OR: 1.177, 95% CI: 1.038–1.335, *P* = 0.011 and OR: 1.148, 95% CI: 1.011–1.304, *P* = 0.033, respectively). A SII value of 876 was the best cut-off value (the area under the curve was 0.791, 95% CI: 0.667–0.915, *P* < 0.05), and the sensitivity and specificity were 73 and 71%, respectively.

**Conclusions:**

The SII and LVEDD are independent prognostic factors for persistent LV systolic dysfunction in patients with PPCM. The SII may be a useful tool for identifying high-risk PPCM patients.

## Introduction

Peripartum cardiomyopathy (PPCM) is a potentially pathogenic disease, usually characterized by heart failure (HF) with decreased ejection fraction in the last month of pregnancy or a few months after delivery (1). Global estimates of the incidence of PPCM vary by region, with reports ranging from 1 in 102 deliveries in Nigeria, to 1 in 10,000 deliveries in Denmark. In the US, its incidence has been estimated at 10.3 per 10,000 live births, and the trend is increasing over time (2). The etiology of PPCM is not clearly known, and it may include possible factors such as inflammation, autoimmune response, imbalance of oxidative stress, induction of antiangiogenic factors, viral infections, and cytokines activation (1, 3).

PPCM can cause serious consequences, including cardiogenic shock, thromboembolism, mechanical circulatory support, cardiac transplantation, and death (1). Although clinical manifestations and outcomes of PPCM vary widely, clinical investigations showed that left ventricular (LV) recovery occurred in 23–66% depending on the study size, race, and follow-up duration. In some studies, almost all recovery of left ventricular function occurred within 6 months after diagnosis, and other studies have also reported delayed recovery of left ventricular function (4). There are many studies to explore the predictors of left ventricular recovery in patients with PPCM. A number of studies have reported that increased left ventricular end-diastolic diameter (LVEDD), decreased baseline left ventricular ejection fraction (LVEF), older age, late diagnosis, black race and elevated inflammatory plasma markers predicted poor prognosis and lower recovery possibility in patients with PPCM (5, 6). However, predicting which patients will have complete left ventricular recovery and which will develop persistent left ventricular systolic dysfunction is still difficult. Therefore, the identification of early prognostic indicators in patients diagnosed with PPCM is very important in risk stratification, prevention of complications and improvement of prognosis.

The systemic immune-inflammation index (SII) is a new biomarker of inflammation, which is calculated as (neutrophil count) × (platelet count)/(lymphocyte count). SII integrates peripheral lymphocyte, neutrophil and platelet counts into one index, to better reflect the balance between inflammation and immunity (7). SII has been proven to be a powerful prognostic indicator of many types of cancer, and it is a useful prognostic index (8). In several studies, SII was found to be a prognostic marker of coronary heart disease (9, 10). However, the prognostic value of SII in PPCM patients has not been proposed before. The purpose of this study was to evaluate the prognostic value of SII in patients with PPCM for the first time.

## Materials and Methods

### Study Population

A total of 61 patients who were diagnosed with PPCM in our tertiary reference center between January 2015 and December 2020 were included in this retrospective analysis. Demographic parameters, laboratory and echocardiogram data of all the patients were reviewed from their patient files, clinical follow-up visits and electronic database. The study protocol was reviewed and approved by the institutional ethics committee in accordance with the Declaration of Helsinki. PPCM was defined as an occurrence of unexplained HF with LVEF <45%, presenting toward the end of pregnancy or in the months after delivery, abortion or miscarriage in previously healthy women (11). All women were at least 18 years of age. The study exclusion criteria were as follows: (1) patients with valvular heart disease, (2) patients with congenital heart disease, (3) patients with ischemic cardiomyopathy, (4) history of malignant tumors and rheumatic diseases. The follow-up duration was at least 6 months after diagnosis of PPCM. A patient will be considered to have high blood pressure if her blood pressure is ≥140/90 mmHg or taking any anti-hypertensive drug.

All patients underwent two-dimensional and M-mode echocardiography, as well as continuous, pulsed and color Doppler echocardiography at the time of diagnosis and the last follow-up visit. Echocardiographic parameters such as LVEF, LVEDD, and left atrium diameter (LAD) were recorded for statistical analysis.

Blood samples were collected at baseline and laboratory tests were performed. The tests included neutrophil, lymphocyte and platelet counts, N-terminal B-type natriuretic peptide (NT-proBNP), erythrocyte sedimentation rate (ESR), low-density lipoprotein cholesterol (LDL-C), etc. SII was calculated as (neutrophil count) × (platelet count)/(lymphocyte count).

Recovery of LV systolic function was defined as the presence of LVEF > 45%, while non-recovery (persistent LV systolic dysfunction) was defined as the presence of LVEF ≤ 45% at last follow-up visit. The second endpoint was defined as composite adverse cardiac events, including cardiac death or hospitalization due to worsening HF.

### Statistical Analysis

Data were analyzed using the SPSS 26.0 Statistical Package Program for Windows (SPSS, Inc., IL, USA). Continuous variables were presented as mean ± standard deviation (SD) or median with interquartile ranges. Categorical variables were presented as frequency and percentage. Kolmogorov–Smirnov test was used to test normality of distribution. Student's *t*-test was used to compare continuous variables between groups for normally distributed variables and Mann–Whitney *U*-test for variables without normal distribution. The Chi-square or Fisher's Exact test was used to compare categorical variables as appropriate. Univariate and multivariate logistic regression analysis was used to assess the capability of the individual variables to predict persistent LV systolic dysfunction. The receiver operating characteristic (ROC) curve analysis was used to establish an optimum cut-off level of admission SII values to predict persistent LV systolic dysfunction. A *p*-value 0.05 was considered statistically significant.

## Results

A total of 61 patients diagnosed with PPCM were enrolled in our study. The mean follow-up period was 40.5 ± 16.3 months. The mean age of diagnosis was 31.8 ± 5.2 years. The proportion of New York Heart Association (NYHA) functional class 2–4 was 7 cases (11.5%), 17 cases (27.9%), and 37 cases (60.6%). Six patients (9.8%) had cardiogenic shock during initial diagnosis. Most of the patients had an onset of PPCM before delivery (36, 59%), and 25 patients (41.0%) presented in the postpartum period, in which 9 cases occurred after spontaneous delivery and 16 cases were after a cesarean section. In terms of the parity, 22 patients (36.1%) were primiparous while 35 patients have had 2 births (57.4%), 3 patients 3 births (4.9%), and 1 patient 4 births (1.6%). Three cases (4.9%) involved twin pregnancies. Among the patients, 30 cases (49.2%) were complicated with gestational hypertension and 6 cases (9.8%) were complicated with diabetes. Most patients received routine treatment of HF, including diuretics, β-blockers, and angiotensin converting enzyme inhibitor/angiotensin receptor blocker (ACEI/ARB). No significant differences were observed between the two groups when using either diuretics, β-blockers or ACEI/ARB. In our study, none of the patients with PPCM were treated with bromocriptine and mechanical circulatory support.

**Figure 1:**
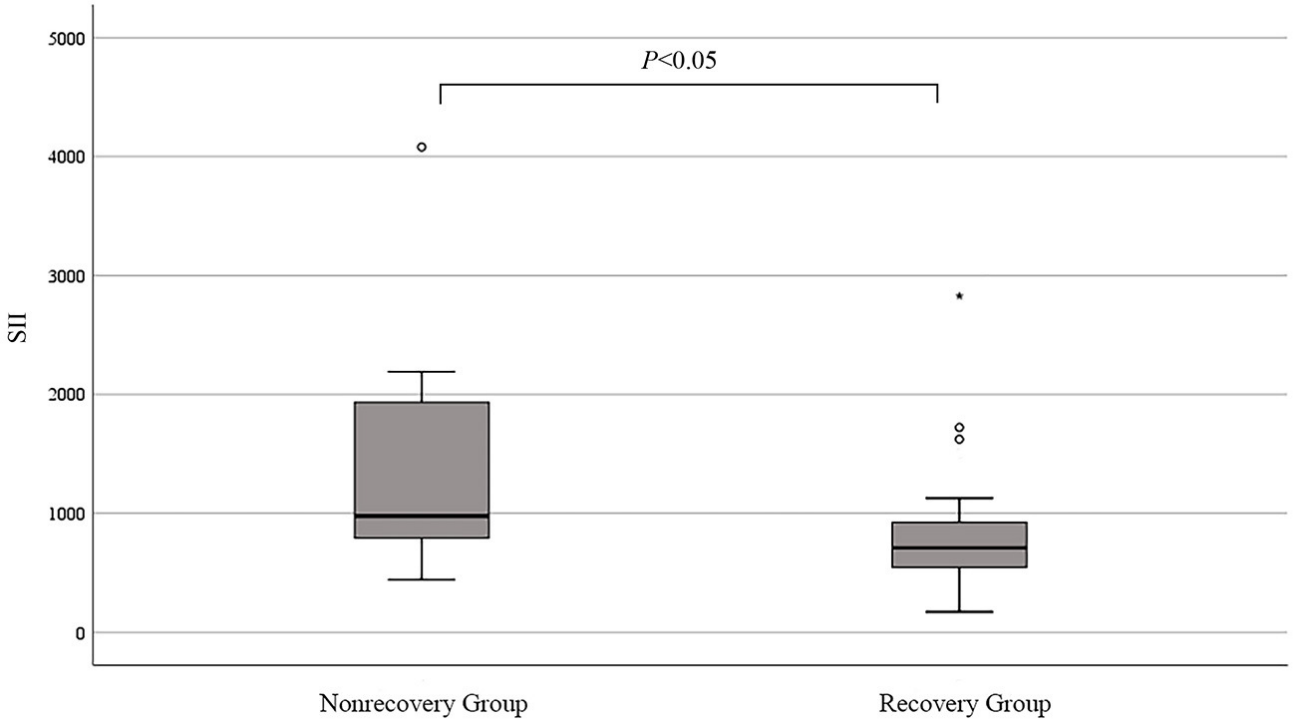
Median SII in non-recovery and recovery groups. °mild outliers; *extreme outliers.

**Figure 2:**
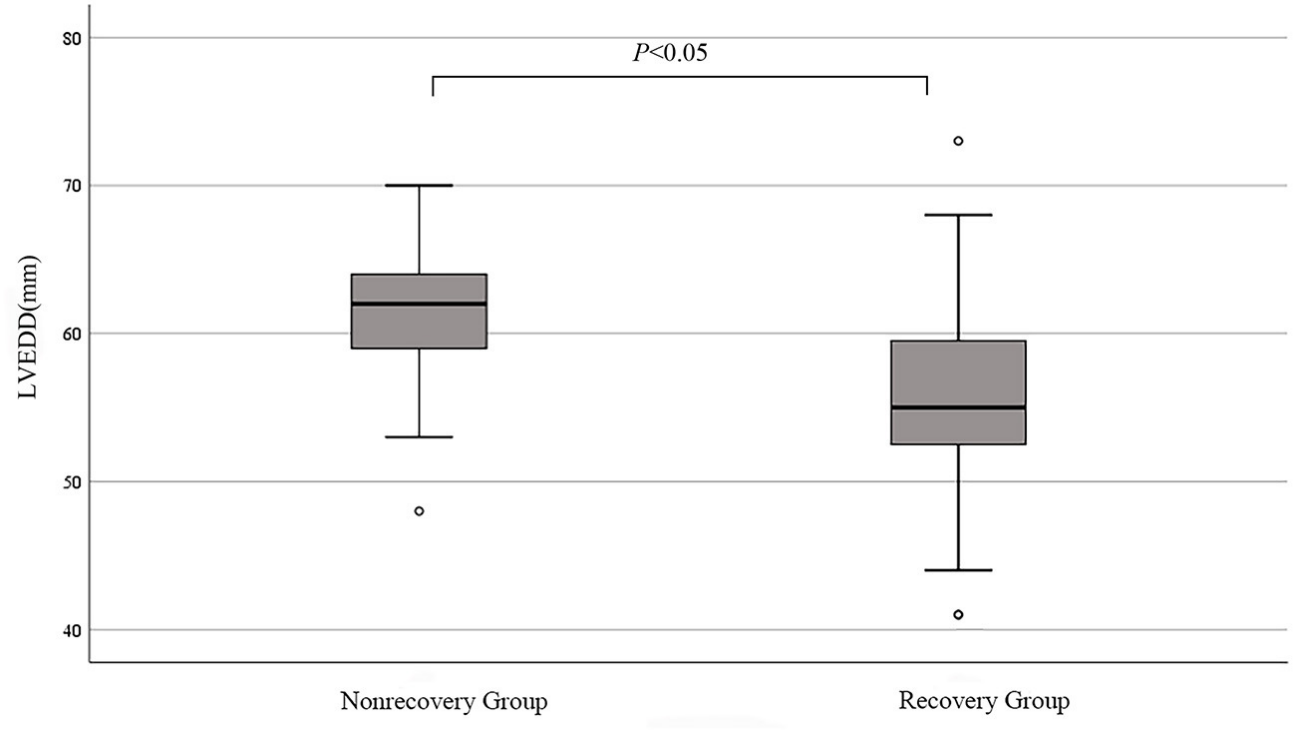
Median LVEDD in non-recovery and recovery groups. °mild outliers.

At the last follow-up visit, 43 patients showed LV function recovery and 18 patients did not at the last follow-up visit. In non-recovery group, 7 patients were re-hospitalized due to worsening HF, 1 patient was dead after ventricular fibrillation. In recovery group, 17 patients (39.5%) were recovered in the first 6 months and 26 patients (60.5%) had LV function recovery more than 6 months. The mean recovery duration was 21.0 ± 16.7 months. After 12–24 months of recovery of LV function, 4 patients stopped the therapy and 18 patients gradually reduced the dose of spironolactone, ACEI/ARB and β-blockers. After 6–24 months of follow-up, there was no significant effect on the LV function after change in therapy in these patients. There were no significant differences in age, cardiogenic shock during initial diagnosis or complications of hypertension between the two groups. The SII of the non-recovery group was significantly higher than that of the recovery group (Figure 1). In addition, LVEDD increased in the non-recovery group (Figure 2). As components of SII calculation, there were no statistical differences in the neutrophil, lymphocyte and platelet counts. Uric acid level in non-recovery group was significantly lower compared to the group with recovery of LV function (*P* = 0.048). Other laboratory parameters and echocardiographic parameters were similar between the two groups (Table 1).

**Table 1:** Baseline clinical, echocardiogram, and laboratory data of patients with and without LV recovery.

	**Recovery group (*n* = 43)**	**Non-recovery group (*n* = 18)**	***P*-value**
Age (year)	32.1 ± 5.0	31.0 ± 5.6	0.436
Presented at postpartum period, *n* (%)	17 (39.5%)	8 (44.4%)	0.722
Hypertension, *n* (%)	24 (55.8%)	6 (33.3%)	0.109
Cardiogenic shock, *n* (%)	2 (4.7%)	4 (22.2%)	0.057
β-blockers, *n* (%)	36 (83.7%)	15 (83.3%)	0.970
ACEI/ARB, *n* (%)	35 (81.4%)	11 (61.1%)	0.176
Spironolactone, *n* (%)	28 (65.1%)	10 (55.6%)	0.680
Systolic blood pressure (mmHg)	132 ± 23	126 ± 25	0.377
BMI (kg/m^2^)	28.09 ± 4.92	30.06 ± 4.99	0.298
NT-proBNP (pg/mL)	3,134 (667–5,510)	5,365 (870–8836)	0.174
Neutrophil (×10^9^/L)	5.66 (3.70–7.46)	7.33 (5.96–8.36)	0.075
Lymphocyte (×10^9^/L)	1.98 ± 0.75	2.07 ± 1.03	0.890
Platelets (×10^9^/L)	256 (188–339)	304 (269–383)	0.071
Monocyte (×10^9^/L)	0.47 (0.33–0.60)	0.51 (0.35–0.70)	0.319
Hemoglobin (g/L)	119 (111–133)	117 (104–124)	0.169
ESR (mm/h)	34.1 ± 19.2	52.7 ± 33.3	0.200
Procalcitonin (ng/mL)	0.19 (0.05–0.40)	0.25 (0.13–0.54)	0.696
D-Dimer (μg/mL)	1.13 (0.62–2.74)	1.01 (0.77–2.22)	0.725
ALT (U/L)	25 (17–38)	25 (14–50)	0.800
Albumin (g/L)	33.2 ± 6.2	35.5 ± 4.0	0.095
Uric acid (μmol/L)	447.1 ± 164.1	335.9 ± 137.6	0.048
Creatinine (μmol/L)	61 (50–75)	60 (52–70)	0.675
Potassium (mmol/L)	4.09 (3.80–4.41)	4.10 (3.74–4.45)	0.843
LDL-C (mmol/L)	3.1 ± 1.1	2.8 ± 0.8	0.448
Homocysteine (μmol/L)	13.2 (10.53–17.00)	13.9 (12.2–17.1)	0.650
LDH (U/L)	342 (268–505)	345 (254–426)	0.712
FT3 (pmol/L)	3.6 (3.0–4.2)	3.3 (2.9–3.9)	0.357
FT4 (pmol/L)	12.4 (10.8–14.9)	13.7 (11.2–14.8)	0.673
CK-MB (ng/mL)	1.7 (0.9–4.1)	1.0 (0.9–1.3)	0.220
LVEF (%)	33 ± 10	28 ± 11	0.065
LAD (mm)	40.7 ± 6.0	41.2 ± 5.6	0.832
LVEDD (mm)	55.7 ± 6.9	61.0 ± 6.6	0.022
SII	710 (545–953)	978 (785–1,953)	0.023
SII>876, *n* (%)	13 (30.2%)	12 (66%)	0.008

*ACEI/ARB, angiotensin converting enzyme inhibitors/angiotensin receptor blocker; BMI, body-mass index; NT-proBNP, N-terminal B-type natriuretic peptide; ESR, erythrocyte sedimentation rate; ALT, Alanine aminotransferase; LDL-C, low-density lipoprotein cholesterol; LDH, lactate dehydrogenase; FT3, free thyroid threeiodine; FT4, free thyroxine; CK-MB, Creatine Kinase Isoenzyme-MB; LVEF, left ventricular ejection fraction; LAD, left atrium diameter; LVEDD, left ventricular end-diastolic dimension; SII, systemic immune-inflammation index*.

Table 2 presents primary and secondary clinical endpoints according to SII values. During follow-up period, patients in the high SII group had a higher incidence of persistent LV systolic dysfunction (48 vs. 16.7%; *P* = 0.008). Secondary endpoint was significantly higher in the group with high SII (24 vs. 5.6%; *P* = 0.044). Cardiac death only happened in the group with high SII (4 vs. 0%). Re-hospitalization due to worsening HF developed more frequently in the high SII group (20 vs. 5.6%).

**Table 2:** Primary and secondary clinical endpoints according to SII.

	**High SII (>876) (*n* = 25)**	**Low SII (<876) (*n* = 36)**	***P*-value**
Persistent LV systolic dysfunction, *n* (%)	12 (48%)	6 (16.7%)	0.008
Second endpoint, *n* (%)	6 (24%)	2 (5.6%)	0.044
Cardiac death, *n* (%)	1 (4%)	0 (0%)	0.410
Re-hospitalized due to worsening HF, *n* (%)	5 (20%)	2 (5.6%)	0.112

The univariate and multivariate logistic regression analysis for the two groups are presented in Table 3. The variables that were significant in the univariate logistic regression analysis (*P* < 0.05) were included in the multivariate analysis. Only SII and LVEDD were identified as independent predictors of persistent LV systolic dysfunction in patients with PPCM (OR: 1.177, 95% CI: 1.038–1.335, *P* = 0.011 and OR: 1.148, 95% CI: 1.011–1.304, *P* = 0.033, respectively).

**Table 3:** Univariate and multivariate logistic regression analysis for non-recovery and recovery groups.

	**OR**	**95%CI**	***P*-value**
**Univariate logistic regression**
Age	0.957	0.859–1.064	0.430
Onset in the postpartum period	1.350	0.442–4.123	0.598
Hypertension	2.200	0.691–7.006	0.182
Parity	0.661	0.262–1.666	0.380
β-blockers	0.972	0.221–4.273	0.970
ACEI/ARB	0.359	0.106–1.216	0.100
Spironolactone	0.670	0.218–2.055	0.483
Systolic blood pressure	0.989	0.966–1.013	0.372
BMI	1.087	0.930–1.271	0.294
Neutrophil	1.178	0.974–1.425	0.092
Lymphocyte	1.049	0.539–2.042	0.888
Platelets	1.004	0.999–1.010	0.132
Hemoglobin	0.973	0.942–1.005	0.093
ESR	1.034	0.994–1.075	0.095
Procalcitonin	0.861	0.494–1.502	0.598
D-Dimer	0.797	0.556–1.141	0.215
Albumin	1.077	0.971–1.195	0.161
LDL-C	0.777	0.410–1.474	0.440
CK-MB	0.930	0.733–1.181	0.548
LVEF	0.948	0.894–1.004	0.070
LAD	1.012	0.907–1.130	0.827
LVEDD	1.127	1.011–1.257	0.031
SII	1.093	1.008–1.184	0.031
**Multivariate logistic regression**
SII	1.177	1.038–1.335	0.011
LVEDD	1.148	1.011–1.304	0.033

*ACEI/ARB, angiotensin converting enzyme inhibitors/angiotensin receptor blocker; BMI, body-mass index; ESR, erythrocyte sedimentation rate; LDL-C, low-density lipoprotein cholesterol; CK-MB, Creatine Kinase Isoenzyme-MB; LVEF, left ventricular ejection fraction; LAD, left atrium diameter; LVEDD, left ventricular end-diastolic dimension; SII, systemic immune-inflammation index*.

**Figure 3:**
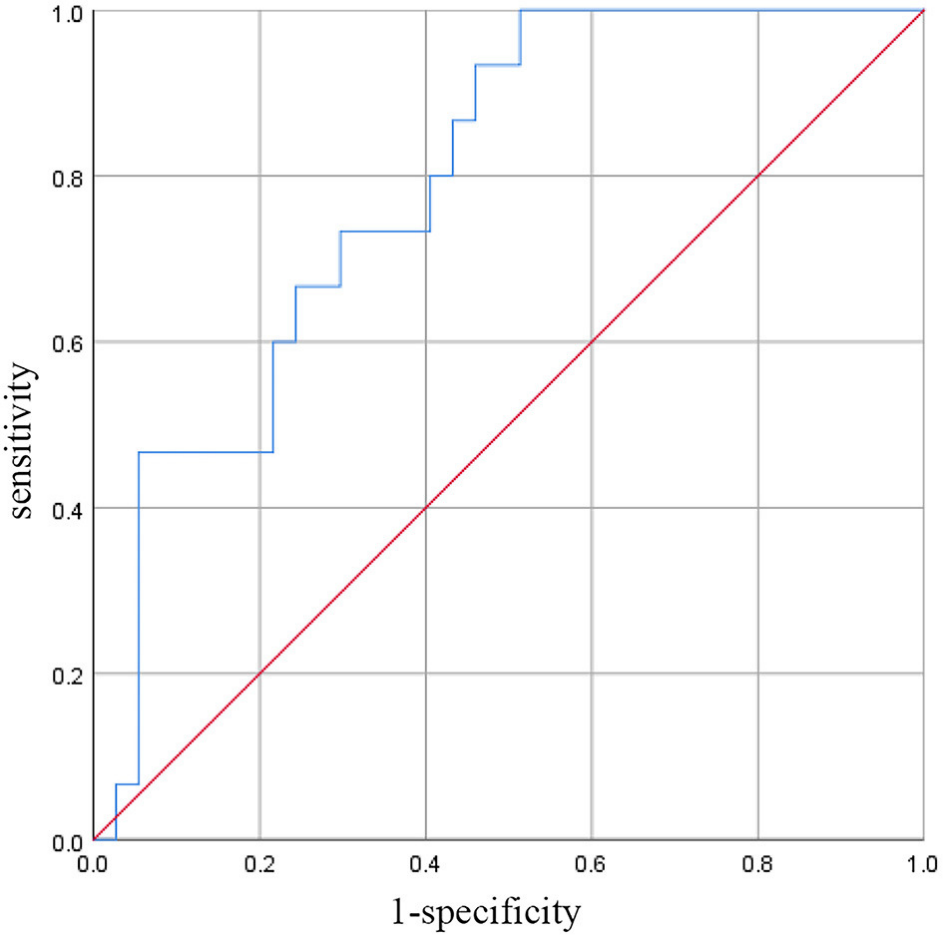
ROC curve of the SII for predicting persistent LV systolic dysfunction.

According to the ROC curve analysis, the best cut-off value was 876. After this level, PPCM patients had a higher rate of persistent LV systolic dysfunction (sensitivity 73%, specificity 71%), as shown in Figure 3. The area under the ROC curve was 0.791 (95% CI: 0.667–0.915, *P* < 0.05).

Table 4 presents baseline clinical, echocardiogram, and laboratory data of recovery group who had recovery within or more than 6 months. There were no significant differences in SII between two groups (*P* = 0.187), but SII in the delayed group has a higher tendency than the other group. Patients with higher NT-proBNP, higher homocysteine (Hcy), and higher lactate dehydrogenase (LDH) were more likely to have delayed recovery of LV function (NT-proBNP: *P* = 0.039, Hcy: *P* = 0.027, LDH: *P* = 0.008).

**Table 4:** Baseline clinical, echocardiogram, and laboratory data of recovery group who had recovery within or more than 6 months.

	** <6 months (*n* = 17)**	**>6 months (*n* = 26)**	***P*-value**
Age (year)	31.2 ± 4.3	32.8 ± 5.4	0.313
Presented at postpartum period, *n* (%)	5 (29.4%)	12 (46.2%)	0.272
Hypertension, *n* (%)	13 (76.5%)	11 (42.3%)	0.059
Systolic blood pressure (mmHg)	137 ± 26	128 ± 22	0.265
BMI (kg/m^2^)	27.38 ± 5.41	29.01 ± 4.23	0.362
NT-proBNP (pg/mL)	1,534 (315–3,487)	4,108 (1,785–6,809)	0.039
Neutrophil (×10^9^/L)	5.55 ± 2.24	6.24 ± 2.84	0.404
Lymphocyte (×10^9^/L)	1.90 (1.50–2.58)	1.80 (1.44–2.56)	0.794
Platelets (×10^9^/L)	203 (158–333)	275 (235–339)	0.062
Monocyte (×10^9^/L)	0.47 (0.33–0.62)	0.47 (0.32–0.59)	0.852
Hemoglobin (g/L)	118 ± 11	121 ± 20	0.633
ESR (mm/h)	46.4 ± 23.1	29.9 ± 16.6	0.098
Procalcitonin (ng/mL)	0.23 (0.08–0.33)	0.10 (0.04–0.89)	0.804
D-Dimer (μg/mL)	1.08 (0.40–2.02)	1.17 (0.75–3.32)	0.376
ALT (U/L)	26 (19–40)	24 (14–37)	0.576
Albumin (g/L)	33.6 ± 6.7	32.9 ± 6.1	0.738
Uric acid (μmol/L)	348.9 ± 99.1	492.9 ± 170.7	0.053
Creatinine (μmol/L)	59 (50–68)	68 (50–87)	0.117
Potassium (mmol/L)	4.11 (3.80–4.64)	4.08 (3.80–4.40)	0.371
LDL-C (mmol/L)	3.1 ± 0.9	3.0 ± 1.2	0.842
Homocysteine (μmol/L)	11.8 (8.3–12.8)	15.4 (11.2–20.2)	0.027
LDH (U/L)	264 (239–309)	388 (294–530)	0.008
FT3 (pmol/L)	3.4 (3.0–4.1)	3.7 (2.9–4.2)	0.967
FT4 (pmol/L)	11.9 (10.9–13.0)	13.1 (10.6–15.8)	0.267
CK-MB (ng/mL)	1.7 (1.1–3.1)	1.5 (0.6–4.4)	0.960
LVEF (%)	36 ± 7	30 ± 10	0.056
LAD (mm)	40 (32–46)	41 (39–44)	0.482
LVEDD (mm)	54.3 ± 4.4	56.9 ± 8.3	0.280
SII	674 (514–807)	801 (565–1,045)	0.187

*BMI, body-mass index; NT-proBNP, N-terminal B-type natriuretic peptide; ESR, erythrocyte sedimentation rate; ALT, Alanine aminotransferase; LDL-C, low-density lipoprotein cholesterol; LDH, lactate dehydrogenase; FT3, free thyroid threeiodine; FT4, free thyroxine; CK-MB, Creatine Kinase Isoenzyme-MB; LVEF, left ventricular ejection fraction; LAD, left atrium diameter; LVEDD, left ventricular end-diastolic dimension; SII, systemic immune-inflammation index*.

## Discussion

In the present study, we found that the SII of the non-recovery group was significantly higher than that of the recovery group, which was an important predictor of LV recovery. ROC analysis showed that the cut-off value of SII for predicting persistent LV systolic dysfunction was 876. As far as we know, this study is the first to determine the long-term prognostic value of SII in patients with PPCM.

PPCM is a potentially life-threatening disease, usually characterized by HF with decreased ejection fraction in the last month of pregnancy or a few months after delivery. In 2010, the Study Group on peripartum cardiomyopathy of the Heart Failure Association (HFA) of the European Society of Cardiology (ESC) defined PPCM as an idiopathic cardiomyopathy occurring toward the end of pregnancy or in the months following delivery, abortion or miscarriage, without other causes for HF, and with a LVEF <45% (11). The incidence in China is one in 346 live births, but that calculation is only based on data from a tertiary reference center, so this subject needs to be studied on a larger scale (12).

Risk factors for PPCM include multiparity and multiple pregnancies, family history, race, smoking, diabetes, hypertension, pre-eclampsia, malnutrition, and age of the mother (4). The etiology of PPCM is uncertain. The possible factors may include inflammation, autoimmune response, imbalance of oxidative stress, induction of antiangiogenic factors, viral infections, and cytokines activation (1, 3). One theory suggests that the oxidative stress-mediated cleavage of the hormone prolactin into 16-kDa prolactin, which is a smaller antiangiogenic subfragment, may drive PPCM by inducing endothelial damage. 16-kDa prolactin can also induce to release endothelial microparticles containing active compounds, such as microRNAs, into the circulation and may subsequently impair cardiomyocyte metabolism and further promote the occurrence of PPCM (13, 14). Studies have shown that partial or complete recovery of left ventricular function occurred in many patients with PPCM. However, PPCM can also cause serious consequences, including cardiogenic shock, thromboembolism, mechanical circulatory support, heart transplants and death (1). There is still no specific and accurate predictor of PPCM cardiac recovery. Some factors that predict the prognosis of patients with PPCM have been proposed before, such as increased NT-proBNP, prolonged QT intervals and sinus tachycardia in electrocardiography (ECG), decreased LVEF, enlarged LV, decreased systolic blood pressure and increased resting heart rate at the time of diagnosis, however this suggestions have not been verified (15–17).

Studies have demonstrated evidence of inflammatory processes and immune responses characterized by cytokine imbalance associated with PPCM (18). Inflammation can be measured with a variety of hematological and biochemical markers. Some studies have shown that increased plasma markers of inflammation and apoptosis at diagnosis were predictors of poor prognosis of PPCM. Some studies found that baseline C-reactive protein (CRP), tumor necrosis factor-α (TNF-α), and interleukin-6 (IL-6) were associated with mortality in patients with PPCM (19, 20). Lymphocyte, neutrophil, and platelet counts are markers that vary with the severity of inflammation and oxidative stress. In recent years, a new index, SII, was proposed based on circulating immune inflammatory cells such as platelets, neutrophils and lymphocytes, which can reflect the balance between the host's inflammatory and immune status better. The SII were thought to be more specific than CRP or the ESR. It is widely reported that SII has been proven to be a powerful prognostic indicator of many kinds of cancer (8). SII has been found to be a prognostic marker of cardiovascular diseases. A number of studies have shown that SII is associated with poor clinical outcomes of various cardiovascular diseases, including acute coronary syndrome, ST segment elevation and non-ST segment elevation myocardial infarction. Studies have shown that a higher SII is independently associated with future risk of cardiac death, non-fatal myocardial infarction, non-fatal stroke or hospitalization due to HF in patients with coronary artery disease (CAD), and it has a better predictive effect on major cardiovascular events after percutaneous coronary intervention (9, 10). SII can also predict the severity of coronary artery stenosis (21). However, the role of SII as a newly available inflammation-based marker in predicting LV recovery in PPCM has not been evaluated. In our study, the SII of the non-recovery group was significantly higher than that of the recovery group. High SII and increased LVEDD were found to be important predictors of predicting persistent LV systolic dysfunction. Our results suggest that this index can be used to determine the risk of adverse outcomes in patients with PPCM and guide the selection of treatment. Uric acid level in non-recovery group was significantly lower compared to the group with recovery of LV function. We think that this is a chance event due to the small sample size of patients and larger research is needed. The SII cannot predict early or delayed recovery of LV function in the present study, and we think this subject needs to be studied on a larger scale. NT-proBNP, Hcy, and LDH were increased in delayed recovery group. NT-proBNP can predict the prognosis of patients with PPCM have been proposed before (15), but the relationship between Hcy, LDH and PPCM have not been reported before. This phenomenon needs to be further researched. From a clinical point of view, as a new predictor of inflammation and oxidative stress, special attention should be given to SII in the initial evaluation of PPCM.

After LV function recovery of PPCM patients, how long the medical therapy should continue is still unknown. In 2019, the Study Group on PPCM of the HFA of the ESC proposed that a combined therapy regimen should be maintained until 12–24 months after full recovery of LV function (11). In our study, the PPCM patients who stopped or gradually reduced the medical therapy 12–24 months of recovery of LV function has not experienced worsening of LV function so far. However, long-term follow-up is necessary to determine further effects on cardiac function.

Our research has some limitations. The main limitation of this study is that our data came from single-center registrations, and the number of patients was relatively small due to the rarity of PPCM. Hence, the statistical power of some observations may be limited. In addition, prolactin and other inflammatory markers, such as IL-6 and TNF-α, were not measured because they are not usually available in daily practice. Another limitation of this study was that SII levels were evaluated only once and changes in SII were not assessed over time during follow-up visits.

## Conclusions

Increased SII and increased LVEDD are independent prognostic factors for persistent LV dysfunction in patients with PPCM. SII may help to identify high-risk patients with PPCM. The advantages of SII include low cost, simple calculation and good repeatability, so it can be widely used to predict the recovery of LV function. However, our findings should be confirmed in prospective, larger research involving other inflammatory biomarkers to clearly explain the exact role of SII in PPCM.

## Data Availability Statement

The raw data supporting the conclusions of this article will be made available by the authors, without undue reservation.

## Author Contributions

YZ: conception and design and data analysis and interpretation. YT and PB: administrative support. YZ, WL, HY, ZC, and CZ: provision of study materials or patients and collection and assembly of data. All authors writing the manuscript and final approval of manuscript.

## Funding

This work was supported by National Key R&D Plan of China [grant number 2017YFC1700502]; National Natural Science Foundation for Young Scientists of China [grant number 82100279]; Natural Science Foundation of Shandong Province [grant numbers ZR2021MH011 and ZR2019QH010]; and ECCM Program of Clinical Research Center of Shandong University [grant number 2021SDUCRCA004]. The funders had no role in the study design, data collection and analysis, the decision to publish or the preparation of the manuscript.

## Conflict of Interest

The authors declare that the research was conducted in the absence of any commercial or financial relationships that could be construed as a potential conflict of interest.

## Publisher's Note

All claims expressed in this article are solely those of the authors and do not necessarily represent those of their affiliated organizations, or those of the publisher, the editors and the reviewers. Any product that may be evaluated in this article, or claim that may be made by its manufacturer, is not guaranteed or endorsed by the publisher.
